# P-417. Rectal Surveillance for Multi-drug Resistant Organisms colonisation Among High-Risk Patients Admitted in a Tertiary-Care Hospital in Greece

**DOI:** 10.1093/ofid/ofae631.618

**Published:** 2025-01-29

**Authors:** Efthymia Protonotariou, Lampros Tampakas, Georgios Meletis, Areti Tychala, Evagelia Pontika, Ειrini Georgopoulou, Konstantinia Bampi, Parthenopi Pantelidou, Petros Trikoupis, Zoe Sereni, Margarita Oikonomou, Haroula Katsanou, Paraskevi Mantzana, Simeon Metalllidis, Lemonia Skoura

**Affiliations:** AHEPA University Hospital, School of Medicine, Aristotle University of Thessaloniki, THESSALONIKI, Thessaloniki, Greece; AHEPA University Hospital, School of Medicine, Aristotle University of Thessaloniki, THESSALONIKI, Thessaloniki, Greece; AHEPA University Hospital, Medical School, Faculty of Health Sciences, Aristotle University of Thessaloniki, Thessaloniki, Greece., Thessaloniki, Thessaloniki, Greece; AHEPA Hospital, Thessaloniki, Thessaloniki, Greece; AHEPA University Hospital, School of Medicine, Aristotle University of Thessaloniki, THESSALONIKI, Thessaloniki, Greece; AHEPA University Hospital, School of Medicine, Aristotle University of Thessaloniki, THESSALONIKI, Thessaloniki, Greece; AHEPA University Hospital, School of Medicine, Aristotle University of Thessaloniki, THESSALONIKI, Thessaloniki, Greece; AHEPA University Hospital, School of Medicine, Aristotle University of Thessaloniki, THESSALONIKI, Thessaloniki, Greece; Cleo, AHEPA Hospital, Thessaloniki, Thessaloniki, Greece; CLEO, AHEPA University Hospital, Thessaloniki, Thessaloniki, Greece; AHEPA University Hospital, School of Medicine, Aristotle University of Thessaloniki, THESSALONIKI, Thessaloniki, Greece; AHEPA University Hospital, School of Medicine, Aristotle University of Thessaloniki, THESSALONIKI, Thessaloniki, Greece; Ahepa University Hospital, Thessaloniki, Thessaloniki, Greece; AHEPA University Hospital, School of Medicine, Aristotle University of Thessaloniki, THESSALONIKI, Thessaloniki, Greece; AHEPA University Hospital, School of Medicine, Aristotle University of Thessaloniki, THESSALONIKI, Thessaloniki, Greece

## Abstract

**Background:**

Antimicrobial Resistance (AMR) is a global urgent health threat that has escalated since the COVID-19 pandemic. Greece is a country with high rates of multi-drug resistant microorganisms (MDROs), including extended-sepctrum β-lactamases (ESBL), carbapenem-resistant Gram-negatives and vancomycin-resistant *EnterococcI* (VRE). In the present study rectal surveillance screening for the above mentioned MDROs was performed among high-risk patients admitted to our hospital.

**Figure:**
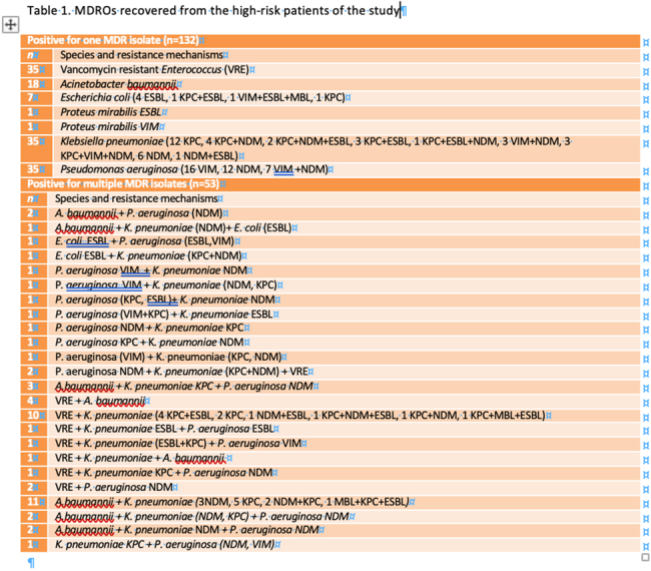

**Methods:**

High risk patients were defined as those previously hospitalized in our or other hospitals, those admitted from rehabilitation centers, oncology, and renal failure patients as well as patients that are already hospitalized in our hospital for more than 30 days. During the study period (Nov 2023 – Apr 2024), 425 patients were screened using Brilliance™ CRE Agar (Oxoid, UK) , Brillliance™ VRE Agar (Oxoid, UK) and CHROMID™ ESBL (bioMérieux, Marcy-l'Étoile, France). Carbapenem-resistant (CR) Enterobacterales and *Pseudomonas aeruginosa* were furtherly tested for carbapenemase production using the NG-Test CARBA 5 immunochromatographic assay (NG-Biotech, France). Isolates grown in the CHROMID™ ESBL plate were tested for ESBL production using ceftazidime and cefotaxime against ceftazidime/clavulanic acid and cefotaxime/clavulanic acid combinations.

**Results:**

185 (43,52%) patients were positive and 240 (56,47%) were negative for the MDROs investigated. Detailed results are shown in Table 1. Among the patients found positive, 89 (48,1%) were from rehabilitation centers, 53 (28,64%) were previously hospitalized elsewhere and 30 (14,05%) were hospitalized in our hospital in previous months. CR *Klebsiella pneumoniae* (n=76), CR *P. aeruginosa* (n=56) and VRE (n=57) were the most common MDROs identified. Fourty-seven patients (25,4%) died, 116 (62,7%) showed improvement and the rest remained stable or moved to other facilities. Among the 240 negatives, 35 (14, 58%) died, 197 (82,08%) were improved and 8 (3,34%) remained stable.

**Conclusion:**

Our results highlight the critical situation regarding the spread of MDR bacteria among hospitalised patients, especially the high-risk ones, showing the imperative need for strengthening the already existing infection control measures.

**Disclosures:**

**All Authors**: No reported disclosures

